# Integrin α_v_β_6_-targeted MR molecular imaging of breast cancer in a xenograft mouse model

**DOI:** 10.1186/s40644-021-00411-9

**Published:** 2021-06-29

**Authors:** Dengfeng Li, Chengyan Dong, Xiaohong Ma, Xinming Zhao

**Affiliations:** 1 Department of Diagnostic Radiology, National Cancer Center/National Clinical Research Center for Cancer/Cancer Hospital, Chinese Academy of Medical Sciences and Peking Union Medical College, No.17, Panjiayuan Nanli, Chaoyang District, Beijing, 100021 China; 2 GE Healthcare, Beijing, 100176 China

**Keywords:** Integrin α_v_β_6_, Ultrasmall superparamagnetic iron oxide (USPIO), Magnetic resonance imaging (MRI), Molecular imaging, RXDLXXL

## Abstract

**Background:**

The motif RXDLXXL-based nanoprobes allow specific imaging of integrin α_v_β_6_, a protein overexpressed during tumorigenesis and tumor progression of various tumors. We applied a novel RXDLXXL-coupled cyclic arginine-glycine-aspartate (RGD) nonapeptide conjugated with ultrasmall superparamagnetic iron oxide nanoparticles (referred to as cFK-9-USPIO) for the application of integrin α_v_β_6_-targeted magnetic resonance (MR) molecular imaging for breast cancer.

**Methods:**

A novel MR-targeted nanoprobe, cFK-9-USPIO, was synthesized by conjugating integrin α_v_β_6_-targeted peptide cFK-9 to N-amino (−NH2)-modified USPIO nanoparticles via a dehydration esterification reaction. Integrin α_v_β_6_-positive mouse breast cancer (4 T1) and integrin α_v_β_6_ negative human embryonic kidney 293 (HEK293) cell lines were incubated with cFK-9-AbFlour 647 (blocking group) or cFK-9-USPIO (experimental group), and subsequently imaged using laser scanning confocal microscopy (LSCM) and 3.0 Tesla magnetic resonance imaging (MRI) system. The affinity of cFK-9 targeting α_v_β_6_ was analyzed by calculating the mean fluorescent intensity in cells, and the nanoparticle targeting effect was measured by the reduction of T2 values in an in vitro MRI. The in vivo MRI capability of cFK-9-USPIO was investigated in 4 T1 xenograft mouse models. Binding of the targeted nanoparticles to α_v_β_6_-positive 4 T1 tumors was determined by ex vivo histopathology.

**Results:**

In vitro laser scanning confocal microscopy (LSCM) imaging showed that the difference in fluorescence intensity between the targeting and blocking groups of 4 T1 cells was significantly greater than that in HEK293 cells (*P* < 0.05). The in vitro MRI demonstrated a more remarkable T2 reduction in 4 T1 cells than in HEK293 cells (*P* < 0.001). The in vivo MRI of 4 T1 xenograft tumor-bearing nude mice showed significant T2 reduction in tumors compared to controls. Prussian blue staining further confirmed that α_v_β_6_ integrin-targeted nanoparticles were specifically accumulated in 4 T1 tumors and notably fewer nanoparticles were detected in 4 T1 tumors of mice injected with control USPIO and HEK293 tumors of mice administered cFK-9-USPIO.

**Conclusions:**

Integrin α_v_β_6_-targeted nanoparticles have great potential for use in the detection of α_v_β_6_-overexpressed breast cancer with MR molecular imaging.

**Supplementary Information:**

The online version contains supplementary material available at 10.1186/s40644-021-00411-9.

## Background

Integrins belong to a heterodimeric transmembrane receptor family that consists of non-covalently associated α and β subunits that integrate the cytoskeleton into the extracellular matrix (ECM), as implied by its name [1]. Among these, integrin α_v_β_3_, an arginine-glycine-aspartate (RGD)-binding integrin, has been considered a promising target for tumor diagnosis and treatment [2–4]. α_v_β_6_ integrin is absent in normal adult epithelia, but is significantly upregulated in various types of cancers of epithelial origin, including colorectal carcinoma [5], breast carcinoma [6], oral squamous carcinoma [7], gastric carcinoma [8], and pancreatic carcinoma [9]. The expression levels of α_v_β_6_ integrin correlate well with tumor progression and metastasis [3, 4, 10], which makes it a promising biological target for molecular imaging for early tumor detection and therapy.

Because of the significance of integrin α_v_β_6_ in tumorigenesis and tumor progression, many pioneering experimental studies have focused on α_v_β_6_-targeted ligands, including peptides and non-peptide inhibitors [11–17]. More recently, a novel sub-nanomolar α_v_β_6_-specific ligand, the nonapeptide cyclo [FRGDLAFp(NMe)K] (referred to as cFK-9) was identified, which is characterized by the RXDLXXL motif sequence [18]. It has high α_v_β_6_ binding affinity (IC_50_ = 0.26 nM), remarkable selectivity against other integrins (α_v_β_3_: IC_50_ = 632 nM; α_5_β_1_: IC_50_ = 73 nM; α_v_β_5_ and α_IIb_β_3_, IC_50_ > 1000 nM), and is very stable in human plasma [18]. Recently, ^68^Ga-labeled cFK-9 has been developed as a radiotracer for in vivo positron emission tomography (PET) imaging of integrin α_v_β_6_ expression, and the radiolabeled probe exhibited specific integrin α_v_β_6_ in vivo imaging of lung cancer [15]. Although promising, nuclear medicine imaging has a relatively low spatial resolution, which may not be conducive to the precise localization of the target lesion. Another limitation is the exposure to ionizing radiation.

Among the various imaging modalities, magnetic resonance imaging (MRI) is increasingly used in the clinical arena due to its exceptional spatial resolution, great soft tissue contrast and lack of ionizing radiation [19, 20]. However, few reports on integrin α_v_β_6_-targeted MR molecular imaging have been published. Magnetic nanoparticles (MNPs), represented by ultrasmall superparamagnetic iron oxide (USPIO) nanoparticles, mainly provide negative T2/T2*-weighted contrast by shortening the T2 relaxation time, leading to the generation of a hypointense signal [21]. Moreover, USPIO nanoparticles have the advantages of an enlarged surface area, good biocompatibility and stability to serve as a vehicle. Various targeting moieties, such as antibodies and peptides, can be conjugated to the surface of USPIO to develop targeted nanoprobes [22, 23].

In the present study, we applied a novel RXDLXXL-coupled peptide conjugated with USPIO nanoparticles (referred to as cFK-9-USPIO) for the realization of integrin α_v_β_6_-targeted in vitro and in vivo MR molecular imaging by using a breast cancer xenograft mouse model.

## Methods

### Synthesis of cFK-9-USPIO

Targeted nanoparticles (cFK-9-USPIO) were prepared based on previous reports [24]. Twenty-nine milligrams of integrin α_v_β_6_ targeted peptides cyclo [FRGDLAFDp(NMe)K] (simplified as cFK-9, Chinese Peptide Company, Hangzhou China) were dissolved in 2.9 mL 0.1 M sodium bicarbonate solution to prepare 0.01 mg μL^− 1^ cFK-9 peptide solution. In addition, 1.4 g N-amino(−NH2)-modified ultrasmall superparamagnetic iron oxide nanoparticles (USPIO) (Sigma-Aldrich, St. Louis, MO, USA) were dissolved in 7.5 mL normal saline and mixed with 2.9 mL cFK-9 peptide solution. The mixture was placed on a shaker (120 rpm) for 8 h at 37 °C, centrifuged in an ultrafiltration tube with a 3-kDa molecular weight cut-off at 12000×g for 10 min, and then washed with saline. The washing step was repeated three times. The final product as cFK-9-USPIO was diluted to 0.1 mg μL^− 1^ using normal pyrogen-free saline.

### Characterizations of the targeted magnetic nanoprobe

Determination of the size of the targeted nanoparticles was performed using a transmission electron microscope (Tecnai F30, FEI Company, Hillsboro, OR, USA). Samples were dispersed in deionized water (30 μg Fe/mL), loaded onto a carbon-coated Cu grid, and imaged at an acceleration voltage of 160 kV. T2-weighted imaging of nanoparticles was performed using a 3.0 T MR scanner (Discovery MR 750, GE Healthcare, Milwaukee, WI, USA) with a T2-weighted fast spin echo pulse sequence (TR = 2050 ms; TE = 85.0 ms; FOV = 80 × 10 mm^2^; data matrix = 256 × 160; slice thickness = 3 ms; spacing = 0.3 mm). The transverse relaxation time (T2) of the nanoprobe was measured at a gradient of Fe concentrations by using the T2 mapping sequence with eight readout echoes (TR = 1700 ms; TE = 9.2, 18.4, 27.6, 36.8, 46, 55.2, 64.4, and 73.6 ms; FOV = 80 × 64 mm^2^; data matrix = 128 × 128; slice thickness = 3 mm; spacing = 0.3 mm; NEX = 2).

### Cell culture

Integrin α_v_β_6_-positive mouse breast cancer cells (4 T1) and integrin α_v_β_6_-negative human embryonic kidney 293 (HEK-293) cells were obtained from the Cell Bank of the Institute of Biochemistry and Cell Biology, Chinese Academy of Science (Shanghai, China). Cells were grown and maintained in RPMI-1640 complete medium supplemented with 10% fetal bovine serum (Gibco, Waltham, MA, USA) at 37 °C in a humidified atmosphere comprising 5% CO_2_ and 95% O_2_.

### In vitro binding assays of targeting peptide cFK-9

To analyze the targeting affinity of the cFK-9 peptide to integrin α_v_β_6_, cellular immunofluorescence was performed. cFK-9 was labeled with AbFlour 647 far-red fluorescent dye (Abbkine, Redlands, CA, USA). 4 T1 and HEK293 cells were cultured in confocal dishes and divided into two groups: an experimental group and a control group. For the experimental group, cells were incubated with AbFlour 647-labeled cFK-9 (0.1 mg μL^− 1^) and excess cFK-9 (100-fold) at 37 °C for 8 h, the control group cells were incubated with AbFlour 647-labeled cFK-9 (0.1 mg μL^− 1^) and PBS solution under the same conditions. The adherent cells were fixed with cold methanol for 10 min and blocked with 5% BSA at 37 °C for 30 min. Hoechst 33342 solution was used to stain the nuclei after incubation with the cells. Fluorescence images were collected using a confocal microscope, as mentioned earlier. All experiments were performed in triplicate. The mean fluorescence intensities of 4 T1 and HEK293 cells were analyzed using Image J bundled with 64-bit Java 1.8.0_112 (https://imagej.nih.gov/ij/download.html).

### In vitro MR imaging

The 4 T1 and HEK293 cells were harvested into 15 mL centrifuge tubes. The cell suspensions were centrifuged at 1006.2×g for 3 min and then washed three times with PBS. cFK-9-USPIO (40 μL) and cFK-9-USPIO with excess cFK-9 (40 μL) solution were added to the cells, and the mixture was incubated for 2 h. Before imaging, the cells were rinsed with PBS twice to remove excess unbound free probes. For MRI, T2-weighted images (T2WI) and T2-mapping were performed using a 3.0 T MR scanner, as mentioned earlier. The reduction of T2 relaxation times was calculated using T2 map (parameters: TR = 1700 ms; TE = 9.4, 18.8, 28.2, 37.6, 47, 56.4, 65.8, and 75.2 ms; FOV = 80 × 64 mm^2^; data matrix = 128 × 128; slice thickness = 3 mm; spacing = 0.3 mm; NEX = 2) to evaluate the targeting effect using in vitro MR imaging. All experiments were performed in triplicate.

### Animals and xenograft tumor model

Animal housing facilities and handling protocols were approved by the Institutional Animal Care and Use Committee of the Cancer Hospital, Chinese Academy of Medical Sciences. The integrin α_v_β_6_-positive 4 T1 tumor and integrin α_v_β_6_-negative HEK293 tumor models were established as previously described [25]. Six-week-old female BALB/c nude mice weighing 20–25 g (*n* = 18) were purchased from Beijing Huafukang Biotechnology Co. Ltd. (Beijing, China). All mice were bred and maintained under specific pathogen-free conditions (22 ± 3 °C, 50–70% relative humidity, and a 12-h light-dark cycle) in accredited animal facilities. Mice (*n* = 12) were injected with 4 T1 cells (5 × 10^6^ cells per mouse) on the left side of the axilla. For the HEK293 model, 5 × 10^6^ HEK293 cells were subcutaneously injected into the left axilla of mice (*n* = 6). The 4 T1 tumor-bearing mice were randomly divided into two groups (6 mice/group). The mice were used when their tumor volumes approached 80–120 mm^3^. All animal experiments were conducted on anesthetized animals and performed according to the guidelines of the Animal Ethics Committee of the Cancer Hospital Chinese Academy of Medical Sciences (NCC2016A002).

### In vivo MR imaging

In vivo MR imaging was performed using a 3.0 Tesla MR scanner (Discovery MR 750, GE Healthcare) equipped with an 8-channel animal coil as described in previous studies [26, 27]. The 4 T1 (*n* = 6) and HEK293 (*n* = 6) tumor-bearing BALB/c nude mice were scanned 0, 1, 8, 12, and 24 h after caudal vein injection with targeted cFK-9-USPIO probes (0.1 mg μL^− 1^, 100 μL/mouse). The same protocol was performed in 4 T1 tumor-bearing mice (*n* = 6) as a non-targeting USPIO control. T2-weighted images (T2WI) were acquired with a T2-fast spin echo sequence (parameters: TR = 1500 ms; TE = 85 ms; FOV = 60 × 10 mm^2^; data matrix = 160 × 160; slice thickness = 1 mm; spacing = 0.8 mm; NEX = 4). The image sequences of the T2-multi-echos map were as follows: TR/TE 1500 ms/7.2, 14.4, 21.6, 28.8, 36, 43.2, 50.4, and 57.6 ms; FOV = 60 × 8 mm^2^; data matrix = 128 × 128; slice thickness = 1 mm; spacing = 0.8 mm; NEX = 4. T2 values of each tumor in 4 T1 and HEK293 mice were calculated from the T2 map at the workstation (Advantage Workstation 4.6; GE Medical System, Milwaukee, WI, USA).

### Histological examination

Tumors and normal tissues of the liver, spleen, kidney, and muscle were collected and fixed in formaldehyde solution at 4 °C for 24 h prior to paraffin embedding. Sections were stained for detection of iron oxide-based nanoparticles using the Perls’ Prussian blue staining method. All tissue sections were stained with hematoxylin and eosin (H&E). In addition, immunohistochemical integrin α_v_β_6_ (Bioss Inc., Beijing, China; 1:200) staining was performed on 4 T1 and HEK293 tumor sections. Subsequently, all the stained sections were observed under a Leica DMI 4000 B fluorescence microscope (Leica, Wetzlar, Germany).

### Statistical analysis

SPSS (version 25.0; SPSS Inc., Chicago, IL, USA) was used for data management and statistical analysis. Data are expressed as mean ± standard deviation (SD) and analyzed by independent-samples Student’s t-test and two-way repeated measures ANOVA for time course analysis, where *P* values less than 0.05 were considered statistically significant.

## Results

### Characterization of cFK-9-USPIO

**Fig. 1:**
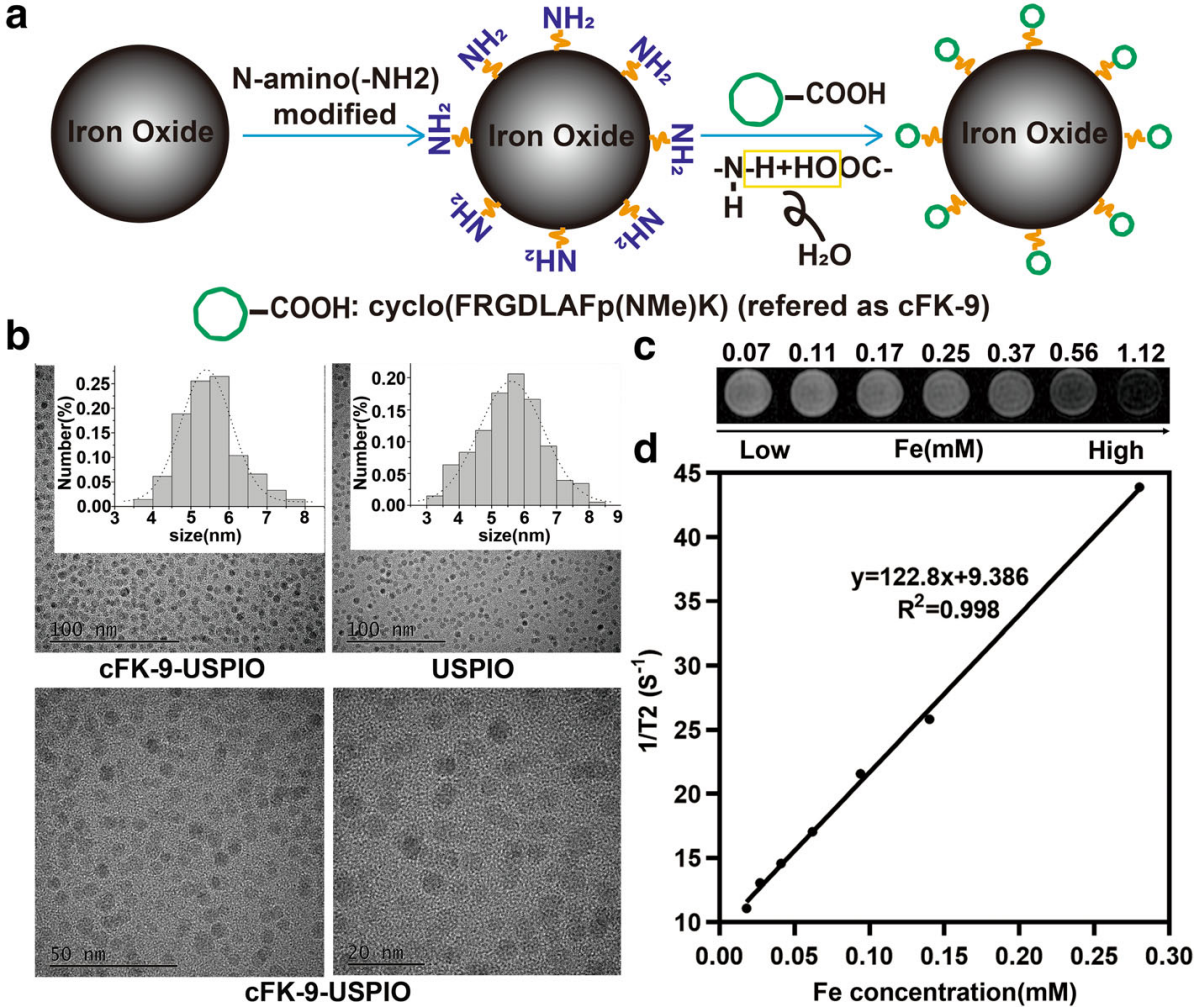
Characterization of the nanoprobe. **a** The schematic synthesis route and structure model of integrin α_v_β_6_-targeted MR nanoprobe (cFK-9-USPIO); **b** The size and histogram analysis of cFK-9-USPIO and USPIO under transmission electron microscope (TEM); **c** T2-Weighted MRI images (3.0 T, Discovery MR 750, GE Medical System, Milwaukee, WI, USA) of increasing cFK-9-USPIO concentrations (mM); D T2 relaxation rate (1/T2, S^− 1^) versus cFK-9-USPIO concentration at 3.0 T

Figure 1A illustrates the synthesis route for cFK-9-USPIO. The TEM images of cFK-9-USPIO and USPIO are shown in Fig. 1B. The core size of cFK-9-USPIO and USPIO determined by TEM was 5.54 ± 1.01 nm and 5.52 ± 0.81 nm, respectively. T2-weighted images at 3.0 T (Fig. 1C) suggested that the T2WI signals of cFK-9-USPIO decreased in an iron concentration-dependent pattern. The cFK-9-USPIO had a short T2 relaxation time and the T2 relaxivity of cFK-9-USPIO was 122.8 mM^− 1^ s^− 1^ (Fig. 1D). In addition, after incubation with 4 T1 cell suspension, the 1:200 M ratio of USPIO to cFK-9 peptides showed the most significant reduction in T2 signal intensity when compared with other different synthetic molar ratio groups (1:10, 1:800, 1:4000), as shown in Supplementary Fig. 1.

### In vitro binding affinity and specificity assays of targeting peptide cFK-9

To examine the integrin α_v_β_6_-targeted binding ability of cFK-9, an in vitro competition binding assay was performed using cellular immunofluorescence. The integrin α_v_β_6_-positive 4 T1 cells and integrin α_v_β_6_-negative HEK293 cells were incubated with AbFlour 647-labeled cFK-9 and AbFlour 647-labeled cFK-9 with excess cFK-9, respectively.

**Fig. 2:**
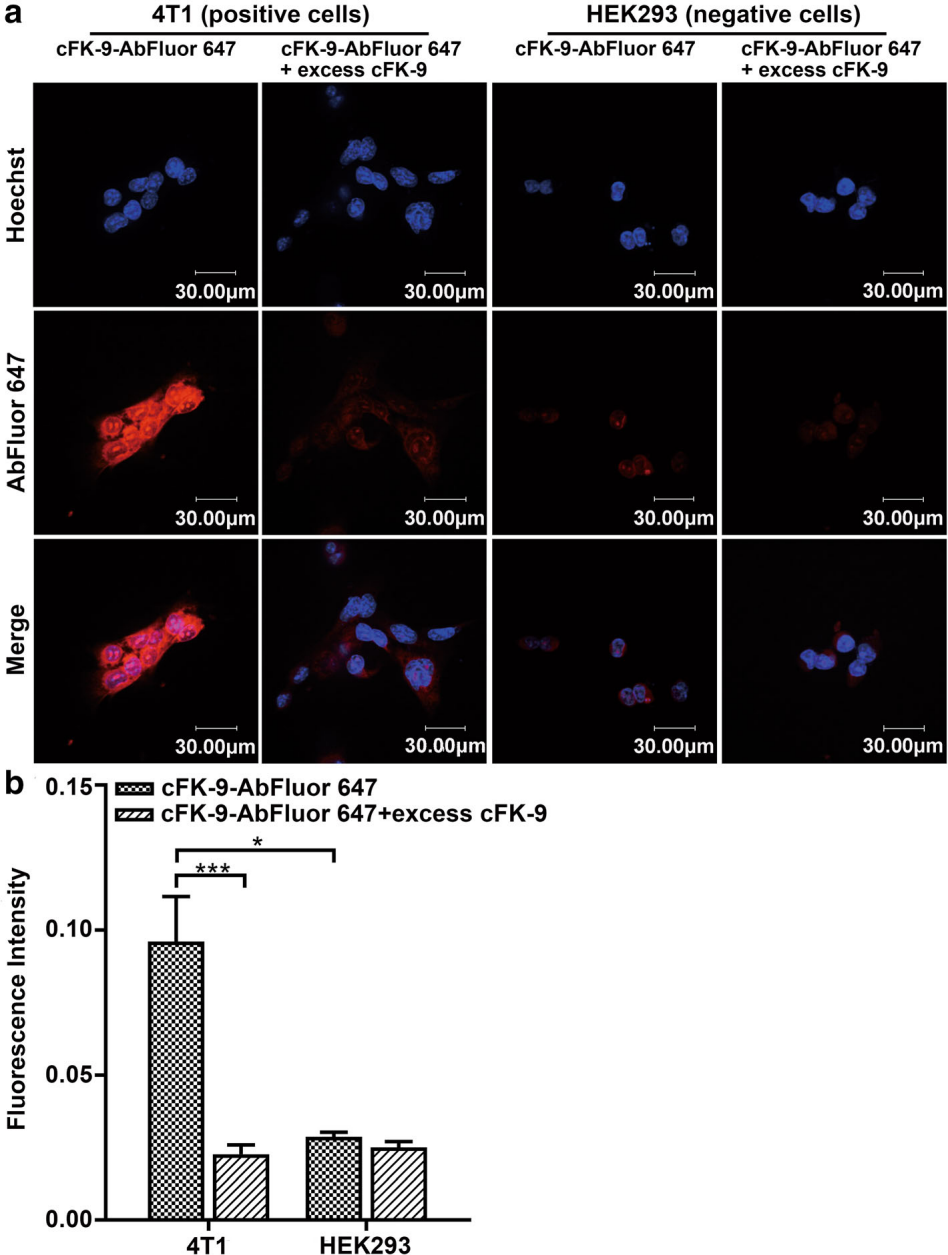
In vitro targeting by laser scanning confocal microscopy (LSCM). **a** The 4 T1 and HEK293 cells were incubated with targeted cFK-9-AbFluor 647 or cFK-9-AbFluor 647 with excess cFK-9 peptides. A lower red fluorescence signal was detected in the 4 T1 membrane incubated with cFK-9-AbFluor 647 with excess cFK-9 peptides compared to cFK-9-AbFluor 647 only. In addition, the red fluorescence of 4 T1 cells incubated with cFK-9-AbFluor 647 is higher than HEK293 cells; **b** Quantitative analysis of 4 T1 and HEK293 cells by Image J bundled with 64-bit Java 1.8.0_112. *, *p* < 0.05; ***, *p* < 0.001

In the 4 T1 cells group, a remarkably lower fluorescent signal was detected in cells incubated with AbFlour 647-labeled cFK-9 and excess cFK-9 compared to cells incubated with AbFlour 647-labeled cFK-9 alone (Fig. 2a). The mean fluorescence intensity was quantitatively analyzed using Image J, and significant differences were observed between fluorescence values after incubation with AbFlour 647-labeled cFK-9 and AbFlour 647-labeled cFK-9 with excess cFK-9 (Fig. 2b). However, no obvious signal intensity changes were detected in HEK293 cells between the AbFlour 647-labeled cFK-9 or AbFlour 647-labeled cFK-9 with excess cFK-9 (Fig. 2b).

As shown in Fig. 2a, 4T1 cells (integrin α_v_β_6_ overexpressed) incubated with AbFlour 647-labeled cFK-9 displayed remarkably higher fluorescence intensity than HEK293 cells (integrin α_v_β_6_ rarely expressed). The mean fluorescence intensity in 4 T1 cells was significantly higher than that in HEK293 cells (Fig. 2b). Similar results were observed for two additional sets of independent experiments.

### In vitro MR imaging of the selective binding of the targeted nanoprobe cFK-9-USPIO to cells

**Fig. 3:**
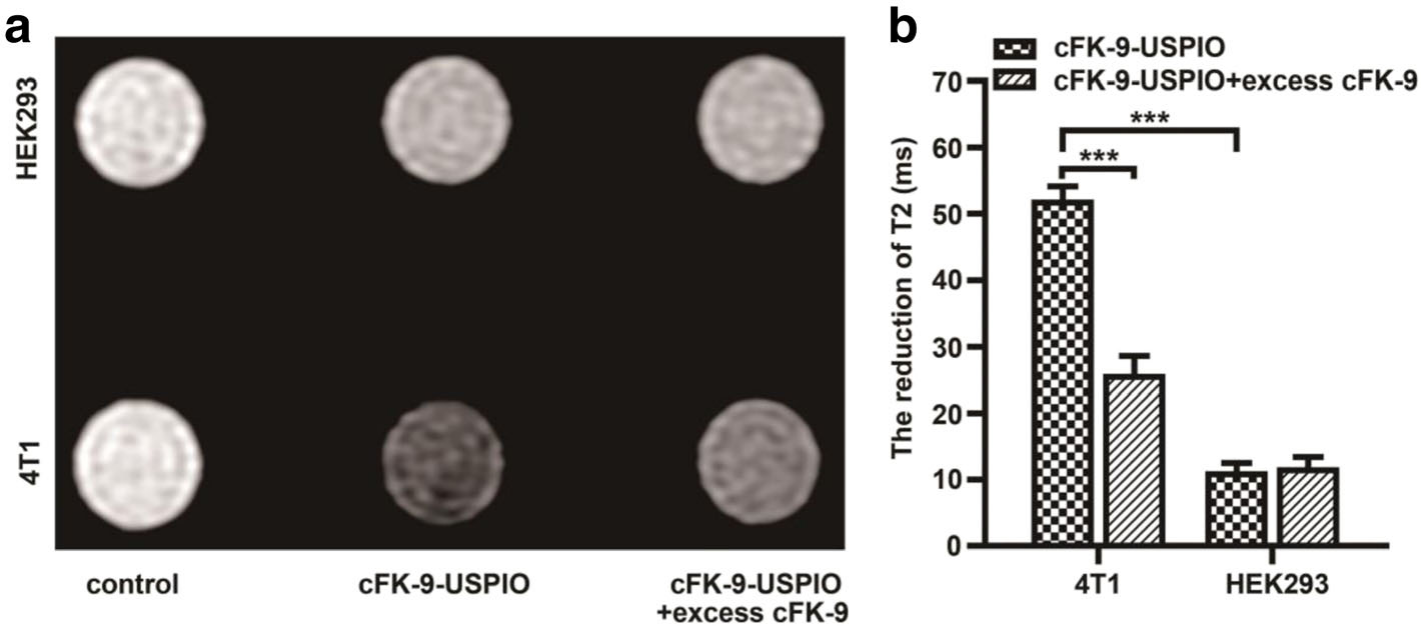
Cytological verification of targeting ability of cFK-9-USPIO nanoparticles using MRI. The harvested 4 T1 and HEK293 cells were incubated with medium containing cFK-9-USPIO or cFK-9-USPIO with excess cFK-9 peptides. **a** MR image shows decreased T2 signal in 4 T1 cell phantoms incubated with cFK-9-USPIO compared to HEK293 cells or control, and higher T2 signal in 4 T1 cell phantoms incubated with cFK-9-USPIO with excess cFK-9 peptides than cFK-9-USPIO only. **b** Quantitative plots of differences in the reduction of T2 values of 4 T1 cells incubated with cFK-9-USPIO compared to that of 4 T1 cells incubated cFK-9-USPIO with excess cFK-9 peptides. In addition, the reduction of T2 values in 4 T1 incubated with cFK-9-USPIO is higher than HEK293. ***, *p* < 0.001

The binding of cFK-9-USPIO to integrin α_v_β_6_-positive 4 T1 cells and integrin α_v_β_6_-negative HEK293 cells was confirmed by in vitro MR imaging. As shown in Fig. 3a, the T2 signal intensity of 4 T1 cells incubated with cFK-9-USPIO was remarkably lower than that of cFK-9-USPIO with excess cFK-9, but no obvious difference in T2 signal was observed in HEK293 cells incubated with cFK-9-USPIO or cFK-9-USPIO with excess cFK-9. The reduction in T2 values in 4 T1 cells incubated with cFK-9-USPIO was significantly higher than that with excess cFK-9 (*P* < 0.001, Fig. 3b). In addition, the reduction of T2 in 4 T1 cells incubated with cFK-9-USPIO was higher than that in HEK293 cells (*P* < 0.001, Fig. 3b). There was no statistically significant difference among the T2 values of 4 T1 and HEK293 cells that were not exposed to the targeted probes (110.18 ± 0.44 vs. 110.47 ± 0.84 ms).

### In vivo MRI of the selective binding of the targeted nanoprobe cFK-9-USPIO to integrin α_v_β_6_-positive 4 T1 tumors

**Fig. 4:**
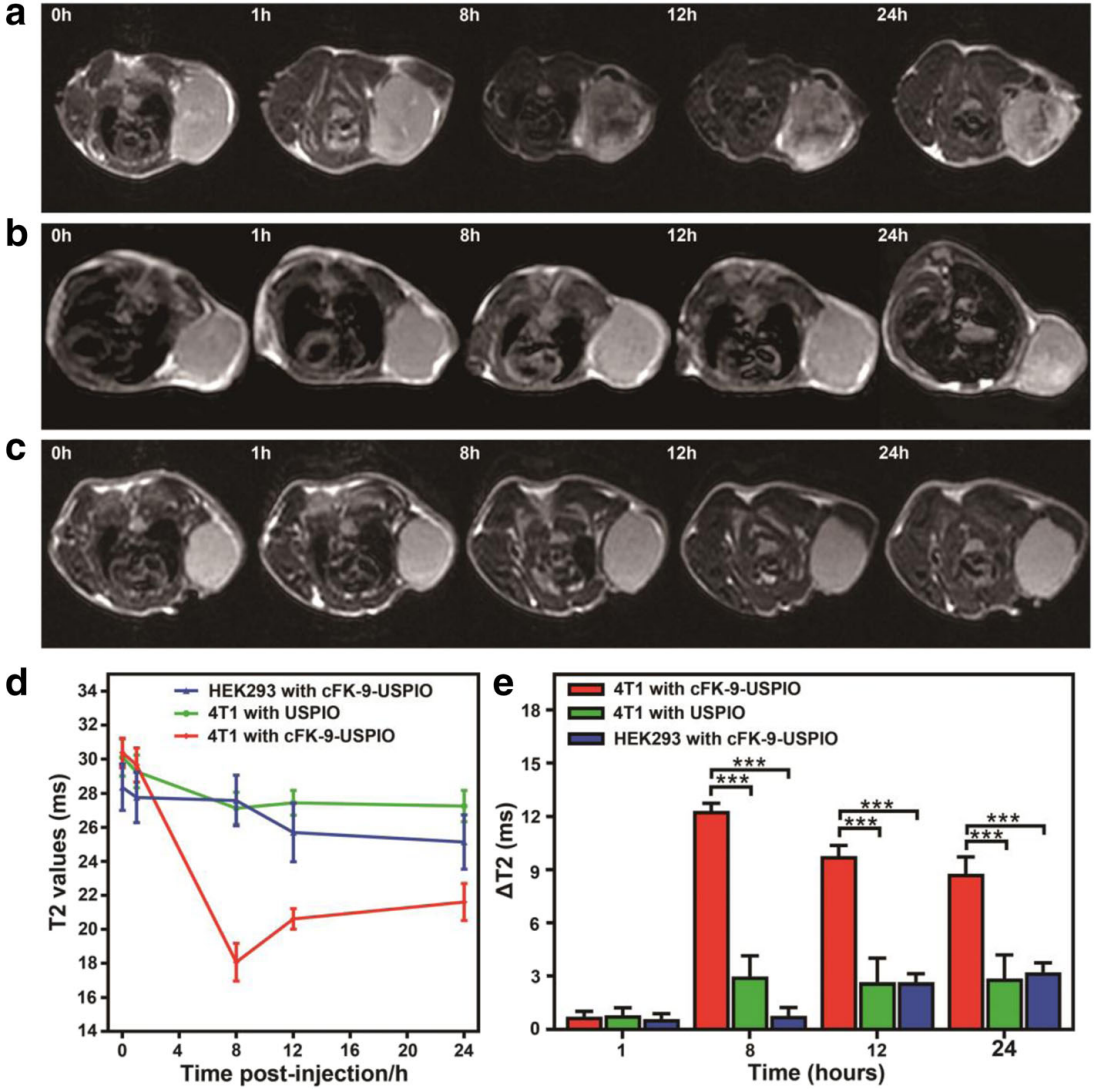
In vivo MRI of xenograft tumor-bearing nude mice. Axial MRI images of the 4 T1 xenograft tumor-bearing nude mice at different time points after tail-vein injection of cFK-9-USPIO (**a**) or USPIO (**b**). An evidently decreased T2 signal was observed in tumor 8 h after administration of cFK-9-USPIO probes through tail vein. In contrast, no obvious T2 signal decrease was found in 4 T1 tumor received USPIO injection. **c** No obvious T2 signal changes in HEK293 tumor after tail-vein injection of cFK-9-USPIO probes. **d** Quantitative T2 values of 4 T1 and HEK293 tumor-bearing mice after cFK-9-USPIO or USPIO injection at different time points. **e** Δ T2 values of 4 T1 tumors received cFK-9-USPIO probe and plain USPIO, and HEK293 tumors received cFK-9-USPIO probes injection at 1 h, 8 h, 12 h, 24 h

The results in Fig. 4 show the MR imaging of mice transplanted with integrin α_v_β_6_-positive 4 T1 and integrin α_v_β_6_-negative HEK293 tumors at different time points after respective injection of cFK-9-USPIO or control USPIO. By comparing the T2-weighted images of tumor-bearing mice at 3.0 T before and after injection with targeted cFK-9-USPIO nanoparticles, we found that T2 signal intensity gradually reduced in 4 T1 tumors after cFK-9-USPIO circulation for 8 h, and the reduced T2 signal effect remained in the tumor area after 24 h. On the contrary, a slight reduction of T2 signal intensity was observed in 4 T1 tumor-bearing mice injected with control USPIO (Fig. 4b) and HEK293 tumor-bearing mice injected with cFK-9-USPIO (Fig. 4c), which illustrated that cFK-9-USPIO was preferentially accumulated in integrin α_v_β_6_-positive tumor tissues. T2 values in 4 T1 tumors remarkably decreased within 8 h of injection with targeted cFK-9-USPIO, and the T2 value reached a minimum at 18.06 ms (Fig. 4d). Further quantitative analysis (Fig. 4e) showed that the ΔT2 values in 4 T1 tumor-bearing mice injected with cFK-9-USPIO were significantly higher than those in 4 T1 mice injected with plain USPIO at 8 h, 12 h and 24 h (12.32 ± 0.44 vs. 2.98 ± 1.16 ms, 9.77 ± 0.61 vs. 2.66 ± 1.34 ms, 8.78 ± 0.94 vs. 2.85 ± 1.34 ms; *P* < 0.001). Significantly higher ΔT2 values were also detected in 4 T1 tumor-bearing mice injected with cFK-9-USPIO than in HEK293 tumor-bearing mice (Fig. 4e, f (1, 10) = 52.462; *P* < 0.001). Interestingly, T2 signals in the liver and spleen (Supplementary Fig. 3) also decreased after 1 h of administration of cFK-9-USPIO and remained decreased until 24 h. On the contrary, no obvious T2 signal intensity changes were detected in the kidney and muscle after injection with cFK-9-USPIO.

**Fig. 5:**
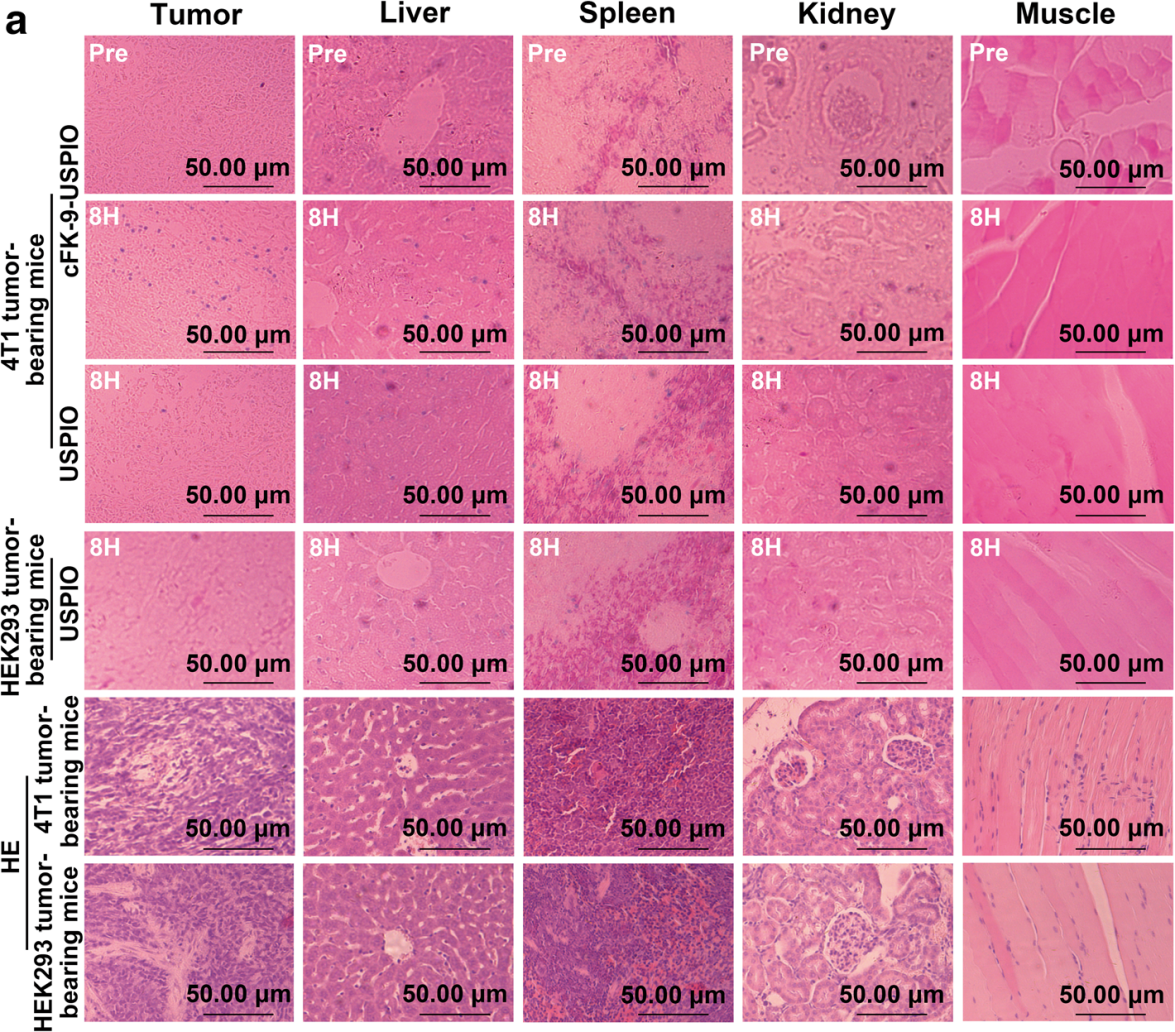
(**a**) Prussian blue, hematoxylin and eosin (H&E) staining of tumor tissues and liver, spleen, kidney, muscle tissues in the 4 T1 and HEK293 xenograft tumor-bearing BALB/c nude mice at 0 h, 8 h after the injection of cFK-9-USPIO or USPIO. A large number of iron blue particles were observed in the 4 T1 tumor tissues but few particles are found in HEK293 tumor tissues from mice injected with cFK-9-USPIO at 8 h. A few blue iron particles are found in liver and spleen tissues of 4 T1 and HEK293 tumor-bearing mice received cFK-9-USPIO at 8 h. For the 4 T1 tumor-bearing mice received USPIO, iron-positive cells were hardly observed in tumor tissues and a few iron blue particles are found in liver and spleen tissues at 8 h. No iron-positive cell is observed in kidney and muscle tissues in 4 T1 neither HEK293 tumor-bearing mice

Prussian blue stained and H&E stained sections of the tumor, liver, spleen, kidney, and muscle of 4 T1 and HEK 293 tumor-bearing mice are shown in Fig. 5.

Prussian blue staining of tissue sections showed high levels of iron positive cells in the tumors of 4 T1 tumor-bearing mice injected with targeted cFK-9-USPIO and fewer iron-containing cells in the tumors of 4 T1 tumor-bearing mice injected with non-targeting USPIO at 8 h. In addition, as shown in Supplementary Fig. 4, low to intermediate levels of iron-containing cells were also observed in the livers and spleens of 4 T1 tumor-bearing mice in both targeted cFK-9-USPIO injection and non-targeting USPIO injection groups at 8 h. In the HEK293 tumor-bearing mice group, low to intermediate levels of iron-positive cells were present in the liver and spleen, but no obvious iron-containing cells were detected in the tumor tissues 8 h after injection with targeted cFK-9-USPIO (Supplementary Fig. 4), which suggested that cFK-9-USPIO specifically targeted the integrin α_v_β_6_-positive tumor. In addition, no tissue damage was detected in H&E-stained sections of the liver, spleen, kidney, and muscle tissues after injection of cFK-9-USPIO (Fig. 5).

### Immunohistochemical (IHC) analysis

As shown in Supplementary Fig. 5, integrin α_v_β_6_ expression in 4 T1 tumor tissues was much higher than that in HEK293 tumor tissues. It is expected that our designed targeted cyclic RGD nonapeptide-conjugated USPIO nanoparticles hold great promise for application in integrin α_v_β_6_ MR molecular imaging.

## Discussion

In this study, we developed a novel RXDLXXL motif-based MR nanoprobe with a simple preparation process and evaluated its imaging capability in vivo. Our findings suggest that integrin α_v_β_6_-targeted nanoparticles specifically bound to integrin α_v_β_6_-positive tumor cells in vitro and selectively accumulated in xenograft tumors, which was detected by in vivo MRI.

Noninvasive visualization and quantification of integrin α_v_β_6_ expression levels in vivo have greatly contributed to the understanding of the mechanisms of tumorigenesis and hold great promise for earlier screening. Recently, integrin α_v_β_6_ was identified as the molecular target in 4 T1 and BxPC3 cells. Expression of α_v_β_6_ correlates with poor patient survival [28]. This study demonstrated that MRI with a novel cFK-9-coupled USPIO could efficiently identify tumors in a 4 T1 xenograft model. The specificity of integrin α_v_β_6_-targeted nanoparticles was investigated in vitro and in vivo. Compared to control plain USPIOs, 4 T1 cells had a higher uptake of cFK-9-USPIO, which could be reduced after competition with uncoupled peptides, which verified the specific binding of cFK-9-USPIO to integrin α_v_β_6_. For in vivo experiments, 4 T1 tumor models were used in which integrin α_v_β_6_ was solely expressed on the tumor, making these models ideal for investigating the potential of DLXXL-based probes to image tumor localization. The results of the present study demonstrated that cFK-9-USPIO showed a heterogeneous signal decrease mainly in the periphery and some central areas of the tumor, which is consistent with the characteristic abundant angiogenesis and integrin α_v_β_6_ expression pattern of the tumor [3]. Quantitative analysis also indicated that the T2 relaxation time decreased significantly more after injection of cFK-9-USPIO than after injection of plain control particles. Taken together, these findings suggest that cFK-9-USPIO is able to specifically and efficiently probe tumorigenesis in a human 4 T1 xenograft model using MRI. The availability of α_v_β_6_-targeted peptides provides an exciting toolbox for targeting different tumor phenotypes.

Compared to other molecular imaging approaches, such as single-photon emission computed tomography (SPECT), positron emission tomography (PET), optical imaging and ultrasound imaging, MR imaging can provide the most exquisite anatomical soft tissue details with high spatial resolution [19, 20]. USPIO nanoparticles have been developed as an imaging contrast agent in a considerable number of studies for MR targeted molecular imaging [26, 29–31]. Upon surfacemodification, the USPIO nanoparticles can be conjugated with various targeting moieties, such as antibodies, peptides, polysaccharides, nucleotides, and ligands, to develop targeted nanoprobes [22, 23]. However, MRI has a relatively low sensitivity compared to nuclear medicine. Therefore, there is an urgent need to increase the selectivity of cFK-9-USPIO MR molecular probes that can accumulate in the target α_v_β_6_-positive tumor cells and induce a significant T2 signal change in the tissue to be detected by MRI. To tackle this problem, different synthetic molar ratio protocols for improving binding affinity were subsequently attempted in our in vitro studies. Our data suggest that the 1:200 ratio of cyclic RGD nonapeptides to USPIO used in synthesis could significantly reduce T2 signal intensity and quantitative T2 values when incubated with 4 T1 cell suspension. In the present study, the core size of cFK-9-USPIO determined by TEM was 5.54 ± 1.01 nm, which allows these nanoparticles to readily penetrate into the target tumor tissue through the compromised vascular endothelium. In addition, our targeted probes cFK-9-USPIO possessed distinct T2 relaxivity (122.8 mM^− 1^ s^− 1^), which could remarkably shorten T2 relaxation time and result in a decrease in T2 signal intensity in MR imaging in the targeted areas.

In our study, integrin α_v_β_6_-expressing 4 T1 cells and α_v_β_6_-negative HEK293 cells were used to establish the positive and negative control models, respectively. Our experimental results showed that the targeted probe successfully reduced T2 signal intensity in the in vitro MRI of integrin α_v_β_6_-positive tumor cell phantoms and the in vivo MRI of α_v_β_6_-expressing tumor xenografts. Moreover, a large number of iron-containing cells were detected by Prussian blue staining of 4 T1 tumor tissues, which further confirmed that our targeted probes cFK-9-USPIO were truly binding to the integrin α_v_β_6_-positive tumor. These data confirmed that targeted cFK-9-USPIO nanoparticles in our in vivo experiments were successfully administered as effective MR contrast agents due to distinct T2 relaxivity, and the coupled cyclic RGD nonapeptides (cFK-9) could mediate targeted binding in integrin α_v_β_6_-positive tumors, so as to achieve MR-targeted imaging at molecular imaging.

Furthermore, significant T2 signal intensity reduction was also observed in the liver and spleen of mice injected with cFK-9-USPIO nanoparticles, and Prussian blue staining detected moderate quantities of iron-positive cells. In contrast, few iron-positive cells were detected in the kidney and muscle tissue sections by Prussian blue staining. These experimental results are consistent with those of previously published studies [26, 32]. This phenomenon could be explained by nanoparticle clearance mediated by macrophages in the liver and spleen. Previous studies have shown that intravenously administered nanoparticles undergo opsonization by interacting with serum proteins and are eventually cleared by the mononuclear phagocytic system (MPS), leading to non-specific accumulation of nanoparticles in organs including the liver and spleen [22, 33]. In addition, a slight reduction in T2 signal intensity and few iron-positive cells in Prussian blue staining of 4 T1 with non-targeted USPIO and HEK293 with targeted USPIO were observed in our experiments. This phenomenon could be explained by the prerequisite of “passive targeting”, driven by the enhanced permeability and retention (EPR) effect. Nanoprobes tend to accumulate in tumor tissues via leaky tumor vasculature and dysfunctional lymphatic drainage, known as the enhanced permeability and retention (EPR) effect, leading to low levels of non-specific accumulation of off-targeted unbound nanoparticles at tumor sites [34, 35]. Due to the non-specific uptake by the reticuloendothelial system and the EPR effect-mediated accumulation of off-targeted unbound nanoparticles, the sensitivity of nanoprobe-targeted imaging is relatively low in comparison with small-molecule radiotracers that are able to more easily penetrate the biological membrane when considering therapy monitoring [36–38]. Enhancing active targeting to achieve a high signal-to-noise ratio is worth exploring in the future.

There are some limitations to our study. First, a subcutaneous transplantation tumor model was used in our in vivo MRI study, which could not reflect the influence of the tumor microenvironment. Second, our targeted probes, as USPIO-based nanoparticles, were nonspecifically cleared by the reticuloendothelial system (RES) to a great extent. Currently, PEGylation and cell membrane-camouflaging are the most common approaches to enhance the biocompatibility of nanoparticles and reduce their non-specific elimination by reticuloendothelial system (RES) uptake [39–42]. Various surface modifications can be applied, including different polymer coatings such as PEG or dextran, or cell-membrane-camouflaged nanoparticles to prevent our targeted nanoparticles from being rapidly recognized by macrophages in the liver and spleen and prolong their blood circulation when administered intravenously in future research. Third, the presence of passive targeting mediated by the EPR effect and nonspecific accumulation of off-targeted unbound nanoparticles in the tumor lead to “noise” background signal reduction, that interferes with quantitative targeting imaging analysis. Improving the efficiency of active targeting in relation to off-targeted unbound nanoprobes is essential for tumor-targeted imaging and precision therapy. Recent studies indicated that high-affinity ligands and smaller sized nanoparticles could help achieve a high level of active targeting and reduce the interference mediated by the EPR effect [43, 44]. This question is worthy of consideration in our subsequent research. Fourth, apart from the single-mode diagnostic utility of MR targeted imaging, the translational potential of iron-based nanoprobes is relatively limited. The molecules of iron-based nanoparticles are relatively large compared to small-molecule targeting probes, leading to a poorer ability to penetrate biological membranes. In addition, the cytotoxicity and immunotoxicity of iron-based nanoprobes limit their application. Designing novel formulations with better biocompatibility and reducing the iron-mediated toxicity may assist in expanding translational applications [21, 34]. Moreover, multimodal imaging that combines different imaging modalities possesses superior ability to detect tumors, and we are currently exploring the feasibility of dual magnetic resonance/fluorescence imaging for targeting integrin α_v_β_6_ at the molecular level.

## Conclusions

In conclusion, we designed novel integrin α_v_β_6_-targeted nanoparticles and verified that these nanoparticles specifically bound to integrin α_v_β_6_-positive tumor cells in vitro and selectively accumulated in xenograft tumors using in vivo MRI approaches. Our findings demonstrate that integrin α_v_β_6_-targeted nanoparticles have great potential for use in the detection of various α_v_β_6_-rich cancers with MR molecular imaging.

## Supplementary Information

**Additional file 1.**

## Data Availability

The MR images and post-processed data used to support the findings of this study are included within the article. Requests for the raw data, after publication of this article, will be available from the corresponding author upon request.

## Abbreviations

BSA: Bovine serum albumin
cFK-9: Cyclo [FRGDLAFp(NMe)K],
FOV: Field of view
HEK293: Human embryonic kidney cell lines 293
H&E: Hematoxylin and eosin
IHC: Immunohistochemical
LSCM: Laser scanning confocal microscopy
MNPs: Magnetic nanoparticles
MPS: Mononuclear phagocytic system
MRI: Magnetic resonance imaging
NEX: Number of excitation
PBS: Phosphate buffer saline
PEG: Polyethylene glycol.
PET: Positron emission tomography
RES: Reticuloendothelial system
RGD: Arginine-glycine-aspartate
RPMI: Roswell park memorial institute
SPECT: Single-photon emission computed tomography
T: Tesla
TE: Echo time
TEM: Transmission electron microscope
TR: Repetition time
T2WI: T2-weighted images
USPIO: Ultrasmall superparamagnetic iron oxide

## References

[1] Hynes RO (2002). Integrins: bidirectional, allosteric signaling machines. Cell..

[2] Nieberler M, Reuning U, Reichart F, Notni J, Wester HJ, Schwaiger M, et al. Exploring the role of RGD-recognizing integrins in cancer. Cancers. 2017;9(9):116. 10.3390/cancers9090116. 10.3390/cancers9090116 PMC5615331 28869579

[3] Niu J, Li Z (2017). The roles of integrin alphavbeta6 in cancer. Cancer Lett.

[4] Koivisto L, Bi J, Hakkinen L, Larjava H (2018). Integrin alphavbeta6: structure, function and role in health and disease. Int J Biochem Cell Biol.

[5] Yang GY, Xu KS, Pan ZQ, Zhang ZY, Mi YT, Wang JS (2008). Integrin alpha v beta 6 mediates the potential for colon cancer cells to colonize in and metastasize to the liver. Cancer Sci.

[6] Desai K, Nair MG, Prabhu JS, Vinod A, Korlimarla A, Rajarajan S (2016). High expression of integrin β6 in association with the rho-Rac pathway identifies a poor prognostic subgroup within HER2 amplified breast cancers. Cancer Med.

[7] Li X, Yang Y, Hu Y, Dang D, Regezi J, Schmidt BL (2003). Alphavbeta6-Fyn signaling promotes oral cancer progression. J Biol Chem.

[8] Zhang ZY, Xu KS, Wang JS, Yang GY, Wang W, Wang JY (2008). Integrin alphanvbeta6 acts as a prognostic indicator in gastric carcinoma. Clin Oncol (Royal College of Radiologists (Great Britain)).

[9] Sipos B, Hahn D, Carceller A, Piulats J, Hedderich J, Kalthoff H (2004). Immunohistochemical screening for beta6-integrin subunit expression in adenocarcinomas using a novel monoclonal antibody reveals strong up-regulation in pancreatic ductal adenocarcinomas in vivo and in vitro. Histopathology..

[10] Allen MD, Marshall JF, Jones JL (2014). alphavbeta6 expression in myoepithelial cells: a novel marker for predicting DCIS progression with therapeutic potential. Cancer Res.

[11] Hausner SH, DiCara D, Marik J, Marshall JF, Sutcliffe JL (2007). Use of a peptide derived from foot-and-mouth disease virus for the noninvasive imaging of human cancer: generation and evaluation of 4-[18F] fluorobenzoyl A20FMDV2 for in vivo imaging of integrin alphavbeta6 expression with positron emission tomography. Cancer Res.

[12] Zhu X, Li J, Hong Y, Kimura RH, Ma X, Liu H (2014). 99mTc-labeled cystine knot peptide targeting integrin alphavbeta6 for tumor SPECT imaging. Mol Pharm.

[13] Liu Z, Liu H, Ma T, Sun X, Shi J, Jia B (2014). Integrin alphavbeta (6)-targeted SPECT imaging for pancreatic Cancer detection. J Nucl Med.

[14] Zhang C, Kimura R, Abou-Elkacem L, Levi J, Xu L, Gambhir SS (2016). A cystine knot peptide targeting integrin alphavbeta6 for photoacoustic and fluorescence imaging of tumors in living subjects. J Nucl Med.

[15] Notni J, Reich D, Maltsev OV, Kapp TG, Steiger K, Hoffmann F (2017). In vivo PET imaging of the Cancer integrin alphavbeta6 using (68) Ga-labeled cyclic RGD Nonapeptides. J Nucl Med.

[16] Liu H, Gao L, Yu X, Zhong L, Shi J, Jia B (2018). Small-animal SPECT/CT imaging of cancer xenografts and pulmonary fibrosis using a (99m)Tc-labeled integrin alphavbeta6-targeting cyclic peptide with improved in vivo stability. Biophys Rep.

[17] Goodman SL, Hölzemann G, Sulyok GAG, Kessler H (2002). Nanomolar small molecule inhibitors for αvβ6, αvβ5, and αvβ3 Integrins. J Med Chem.

[18] Maltsev OV, Marelli UK, Kapp TG, Di Leva FS, Di Maro S, Nieberler M (2016). Stable peptides instead of stapled peptides: highly potent alphavbeta6-selective integrin ligands. Angewandte Chemie (International ed in English).

[19] Weissleder R, Pittet MJ (2008). Imaging in the era of molecular oncology. Nature..

[20] Chen C, Wu CQ, Chen TW, Tang MY, Zhang XM (2015). Molecular imaging with MRI: potential application in pancreatic Cancer. Biomed Res Int.

[21] Dadfar SM, Roemhild K, Drude NI, von Stillfried S, Knuchel R, Kiessling F (2019). Iron oxide nanoparticles: diagnostic, therapeutic and theranostic applications. Adv Drug Deliv Rev.

[22] Kiessling F, Mertens ME, Grimm J, Lammers T (2014). Nanoparticles for imaging: top or flop?. Radiology..

[23] Yan H, Zhao L, Shang W, Liu Z, Xie W, Qiang C (2017). General synthesis of high-performing magneto-conjugated polymer core–shell nanoparticles for multifunctional theranostics. Nano Res.

[24] Jia Z, Song L, Zang F, Song J, Zhang W, Yan C (2016). Active-target T1-weighted MR imaging of tiny hepatic tumor via RGD modified ultra-small Fe3O4 Nanoprobes. Theranostics..

[25] Jiang X, Du Y, Meng X, Zhang H, Zhao D, Zhao L (2018). Low-dose radiation enhanced inhibition of breast tumor Xenograft and reduced myocardial injury induced by doxorubicin. Dose Response.

[26] Chen X, Zhou H, Li X, Duan N, Hu S, Liu Y (2018). Plectin-1 targeted dual-modality nanoparticles for pancreatic cancer imaging. EBioMedicine..

[27] Wang Q, Yan H, Jin Y, Wang Z, Huang W, Qiu J (2018). A novel plectin/integrin-targeted bispecific molecular probe for magnetic resonance/near-infrared imaging of pancreatic cancer. Biomaterials..

[28] Elayadi AN, Samli KN, Prudkin L, Liu YH, Bian A, Xie XJ (2007). A peptide selected by biopanning identifies the integrin alphavbeta6 as a prognostic biomarker for nonsmall cell lung cancer. Cancer Res.

[29] Kang T, Li F, Baik S, Shao W, Ling D, Hyeon T (2017). Surface design of magnetic nanoparticles for stimuli-responsive cancer imaging and therapy. Biomaterials..

[30] Shi J, Wang F, Liu S (2016). Radiolabeled cyclic RGD peptides as radiotracers for tumor imaging. Biophys Rep.

[31] Jiang Y, Liu S, Zhang Y, Li H, He H, Dai J (2017). Magnetic mesoporous nanospheres anchored with LyP-1 as an efficient pancreatic cancer probe. Biomaterials..

[32] Cui Y, Zhang C, Luo R, Liu H, Zhang Z, Xu T (2016). Noninvasive monitoring of early antiangiogenic therapy response in human nasopharyngeal carcinoma xenograft model using MRI with RGD-conjugated ultrasmall superparamagnetic iron oxide nanoparticles. Int J Nanomedicine.

[33] Sun W, Hu Q, Ji W, Wright G, Gu Z (2017). Leveraging physiology for precision drug delivery. Physiol Rev.

[34] Shah A, Dobrovolskaia MA (2018). Immunological effects of iron oxide nanoparticles and iron-based complex drug formulations: therapeutic benefits, toxicity, mechanistic insights, and translational considerations. Nanomedicine.

[35] Tahmasbi Rad A, Chen CW, Aresh W, Xia Y, Lai PS, Nieh MP (2019). Combinational effects of active targeting, shape, and enhanced permeability and retention for Cancer Theranostic Nanocarriers. ACS Appl Mater Interfaces.

[36] Teirlinck E, Xiong R, Brans T, Forier K, Fraire J, Van Acker H (2018). Laser-induced vapour nanobubbles improve drug diffusion and efficiency in bacterial biofilms. Nat Commun.

[37] Muir RK, Zhao N, Wei J, Wang YH, Moroz A, Huang Y (2019). Measuring dynamic changes in the labile Iron Pool in vivo with a reactivity-based probe for positron emission tomography. ACS Central Sci.

[38] Malhotra S, Dumoga S, Joshi A, Mohanty S, Singh N (2021). Polymeric micelles coated with hybrid nanovesicles enhance the therapeutic potential of the reversible topoisomerase inhibitor camptothecin in a mouse model. Acta Biomater.

[39] Gheibi Hayat SM, Bianconi V, Pirro M, Sahebkar A (2019). Stealth functionalization of biomaterials and nanoparticles by CD47 mimicry. Int J Pharm.

[40] Li S, Liu J, Sun M, Wang J, Wang C, Sun Y (2020). Cell membrane-camouflaged Nanocarriers for Cancer diagnostic and therapeutic. Front Pharmacol.

[41] Wang G, Song L, Hou X, Kala S, Wong KF, Tang L (2020). Surface-modified GVs as nanosized contrast agents for molecular ultrasound imaging of tumor. Biomaterials..

[42] Li M, Fang H, Liu Q, Gai Y, Yuan L, Wang S (2020). Red blood cell membrane-coated upconversion nanoparticles for pretargeted multimodality imaging of triple-negative breast cancer. Biomaterials Sci.

[43] Wang L, Huang J, Chen H, Wu H, Xu Y, Li Y (2017). Exerting enhanced permeability and retention effect driven delivery by ultrafine Iron oxide nanoparticles with T (1)-T (2) switchable magnetic resonance imaging contrast. ACS Nano.

[44] Xu Y, Wu H, Huang J, Qian W, Martinson DE, Ji B (2020). Probing and enhancing ligand-mediated active targeting of tumors using Sub-5 nm ultrafine Iron oxide nanoparticles. Theranostics..

