# Supplementary material for: Integrin αvβ6-targeted MR molecular imaging of breast cancer in a xenograft mouse model
Source: Cancer Imaging. 2021 Jun 29;21:44. doi: 10.1186/s40644-021-00411-9 (PMC8244136; doi:10.1186/s40644-021-00411-9)
Supplement: Supplementary file 1 — Additional file 1. [file 40644_2021_411_MOESM1_ESM.zip › Supplementary Material.docx]

**Supplementary Material**

**Integrin αvβ6-targeted MR molecular imaging of breast cancer in a xenograft mouse model**

Dengfeng Li^1^, Chengyan Dong^2^, Xiaohong Ma^1^, Xinming Zhao^1^

^1^Department of Diagnostic Radiology, National Cancer Center/National Clinical Research Center for Cancer/Cancer Hospital, Chinese Academy of Medical Sciences and Peking Union Medical College, Beijing, 100021, China

^2^GE Healthcare, Beijing, China, 100176

**Methods**

Immunofluorescence staining of 4T1 and HEK293 cells

Confocal laser scanning microscopy was used to observe integrin α_v_β_6_ on the 4T1 and HEK293 cell membrane. Briefly, 4T1 and HEK293 cells were grown on confocal dishes and fixed with cold methanol for 20 min. This was followed by incubated with blocking buffer contained 5% BSA for 30 min to suppress unspecific binding sites. After that, the cells were stained with anti-integrin α_v_β_6_ antibody (1:100 dilution in PBS with 1% BSA; Abcam, Cambridge, UK) for 1 h at room temperature and incubated with Alexa Fluor 488 (AF488)-conjugated anti-mouse IgG (H + L) F(ab')_2_ fragment secondary antibody (1:1000 dilution; Cell Signaling Technology Inc., MA, USA) for 30 min. Hoechst 33342 solution was used for nuclei staining at room temperature for 15min. Cell samples were examined using a confocal laser-scanning microscope (UltraView VOX; PerkinElmer, Waltham, MA, USA).

**Results**

Immunofluorescence staining of 4T1 and HEK293 cells

Immunofluorescence staining was performed to evaluate the expression of integrin α_v_β_6_ on the 4T1 and HEK293 cell membrane. As shown in Supplemental Figure 2A, a stronger green fluorescent signal was detected in 4T1 membrane and a fainter signal was observed in HEK293. Significant differences between mean fluorescence values were observed in the 4T1 and HEK293 cells incubated with AF488-labeled secondary antibody (Supplemental Figure 2B), which indicated integrin αvβ6 was expressed on 4T1 cell membrane, no expression was observed on HEK293 cell membrane. Therefore, the integrin α_v_β_6_-positive 4T1 cells and integrin α_v_β_6_-negative HEK293 cells were used in the following *in vitro* and *in vivo* imaging experiments. Similar results were observed in two additional sets of independent experiments.


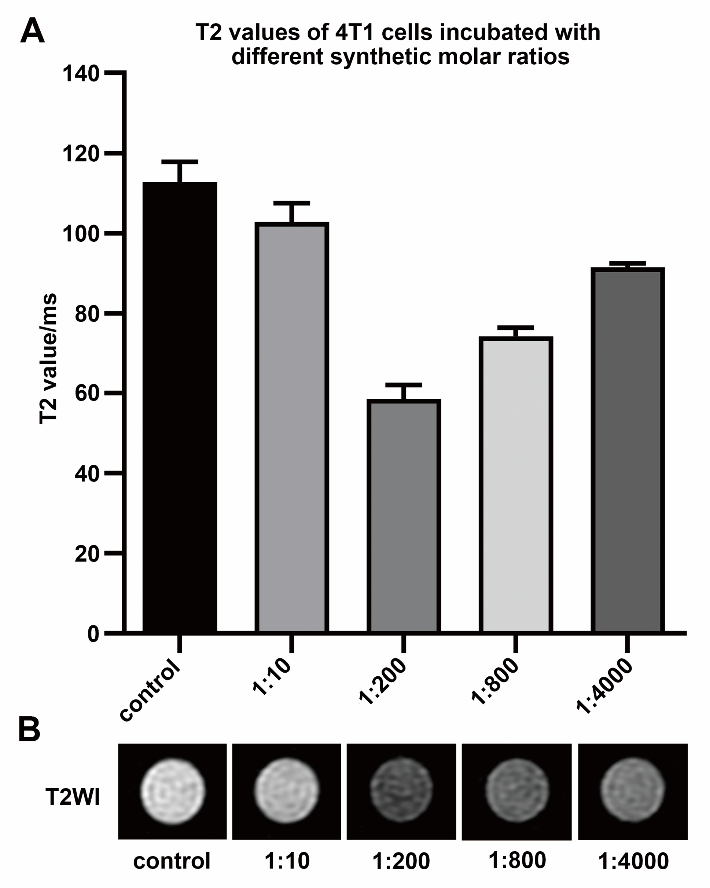


Supplementary Figure **1.** T2 values and T2-weighted images of 4T1 cells incubated with nanoprobes of different synthetic molar ratios.


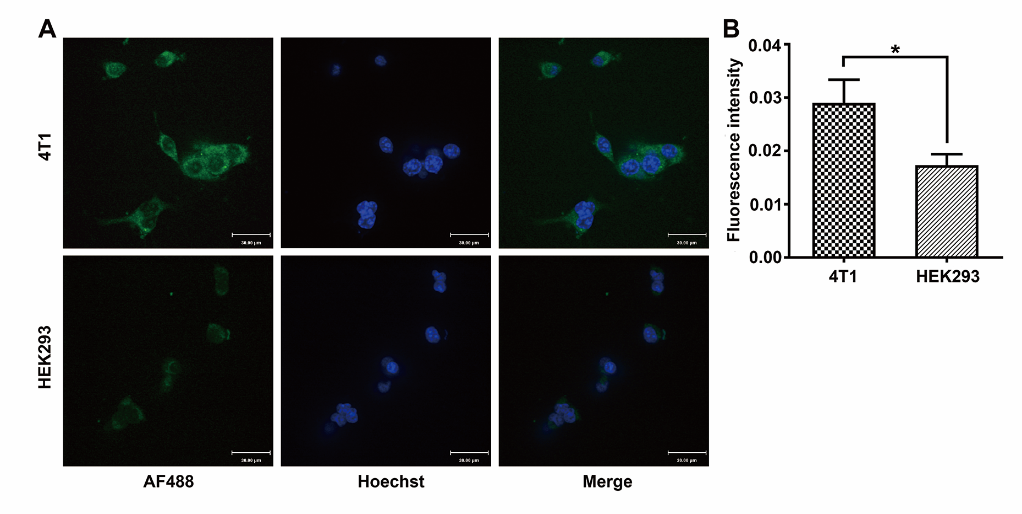


Supplementary Figure **2.** Detection of cell membrane expression integrin α_v_β_6_ by immunofluorescence. (A) Laser-scanning confocal microscopy (LSCM) imaging of 4T1 and HEK293 cells incubated with cFK-9-Alexa Fluor 488, the green fluorescence of 4T1 incubated with cFK-9-Alexa Fluor 488 was significant higher than HEK293. (B) Mean fluorescence intensity of 4T1 and HEK293 cells was quantitatively analyzed by Image J bundled with 64-bit Java 1.8.0_112. * *p* < 0.05.


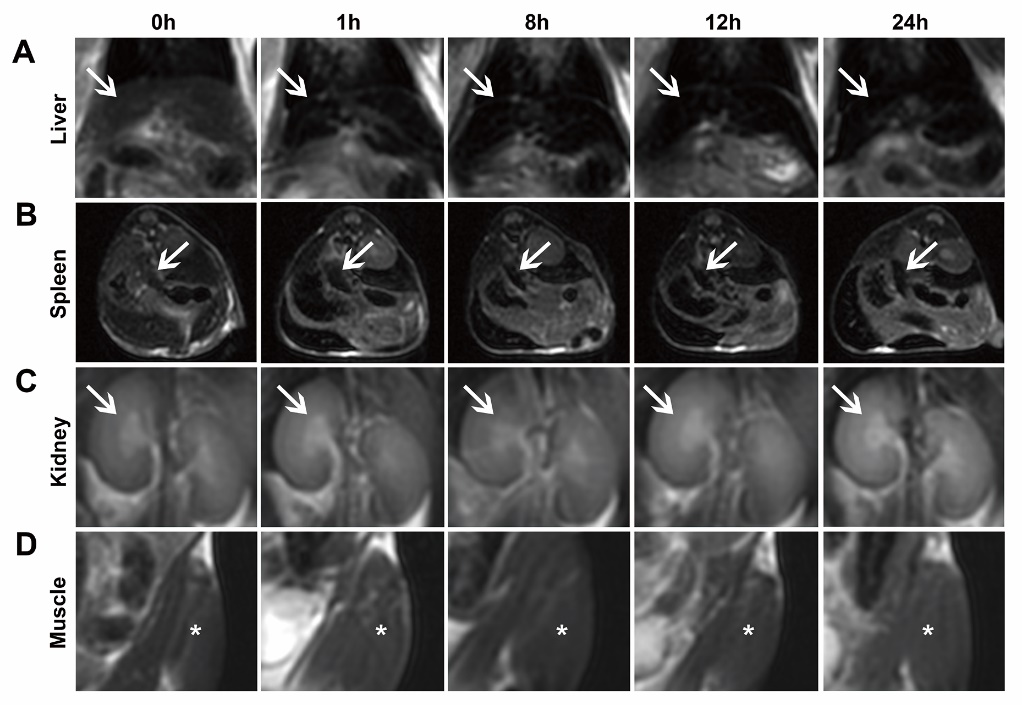


Supplementary Figure **3.** T2-weighted MR imaging of liver, spleen, kidney and muscle in 4T1 xenograft tumor-bearing BALB/c nude mice at varied time points after injection of cFK-9-USPIO. (A) and (B) showed reduced T2 signals in the liver and spleen (white arrow) of 4T1 mice from 1 h after injection with cFK-9-USPIO until 24 h compared with pre-injection images. (C & D) No obvious T2 signal changes were observed in the kidney and muscle of mice post-injection with cFK-9-USPIO.


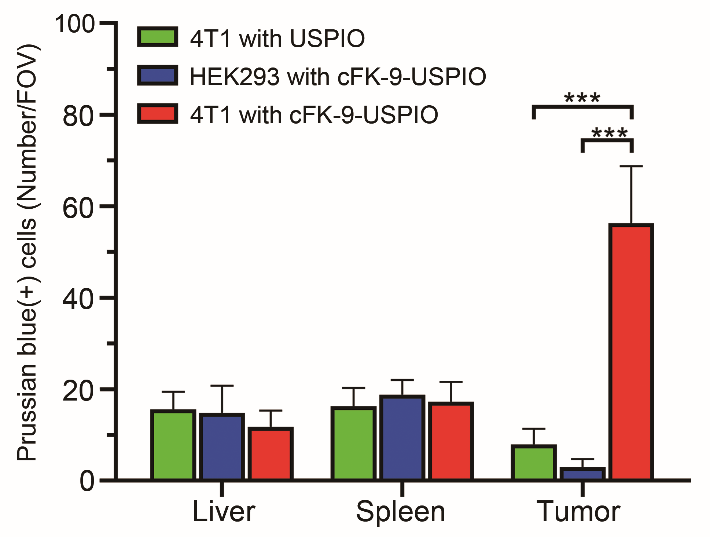


Supplementary Figure **4.** Prussian blue (+) positive cell count in the liver, spleen and tumor sections. Quantitative data showed that Prussian blue positive cells were lowly to immediately distributed in the liver and spleen tissues after injected with cFK-9-USPIO and plain USPIO at 8 h. Iron positive cells were significantly higher in tumor of 4T1 mice received cFK-9-USPIO for 8 h. ***, *p* < 0.001.


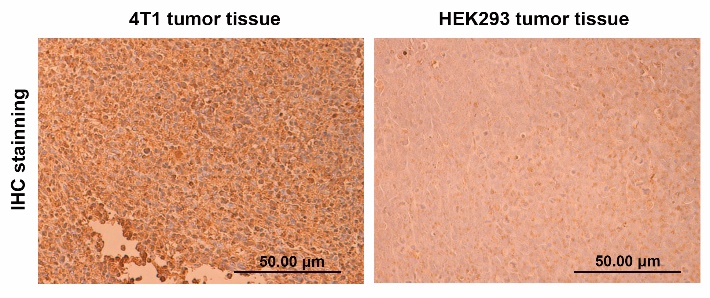


Supplementary Figure **5.** Immunohistochemical results of 4T1 and HEK293 tumors. integrin α_v_β_6_-positive cells are brown.
