# Supplementary figures and images for: Integrin αvβ6-targeted MR molecular imaging of breast cancer in a xenograft mouse model
Source: Cancer Imaging. 2021 Jun 29;21:44. doi: 10.1186/s40644-021-00411-9 (PMC8244136; doi:10.1186/s40644-021-00411-9)

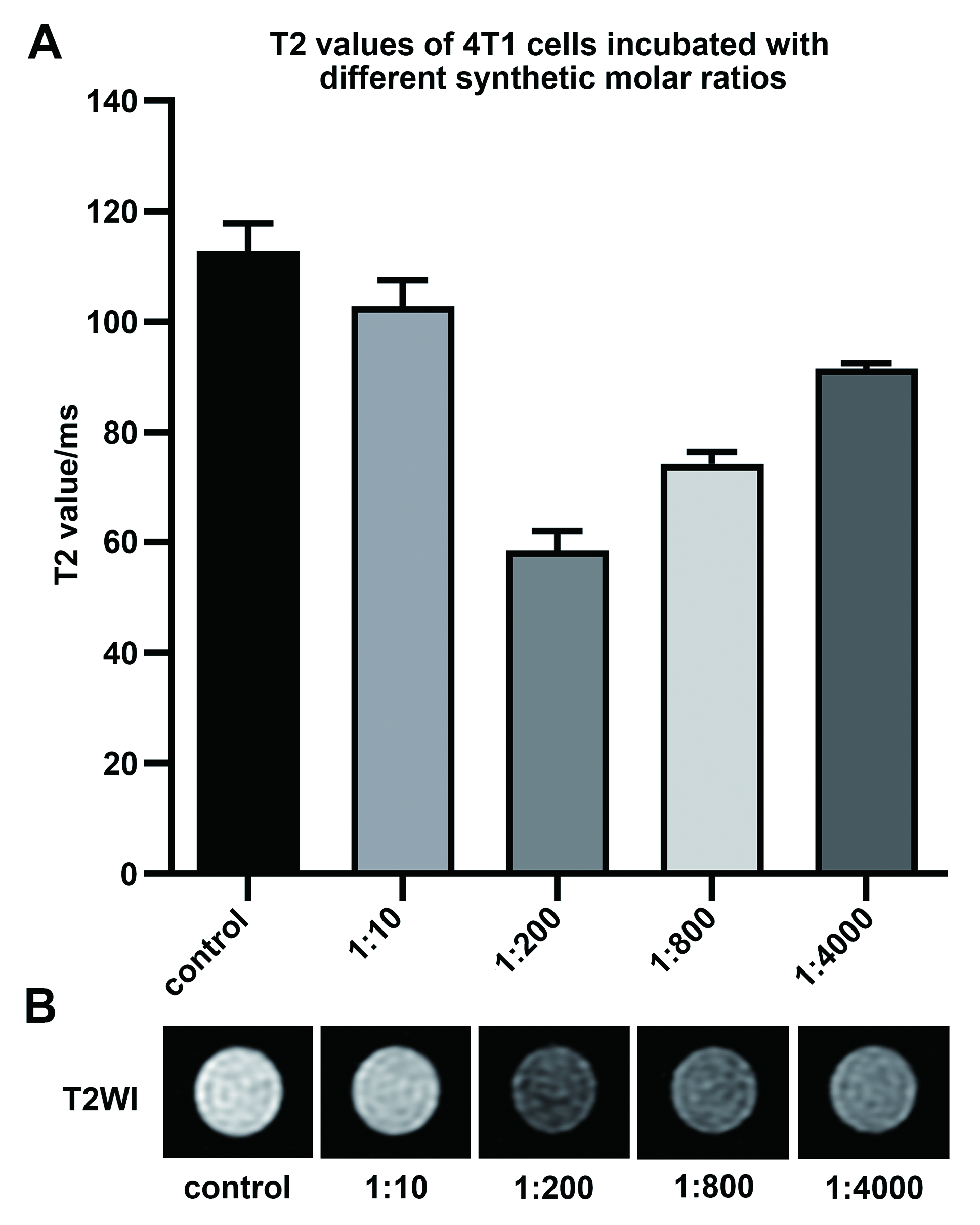

Supplement: Supplementary file 1 — Additional file 1. [file 40644_2021_411_MOESM1_ESM.zip › Supplementary Figure1.tif]

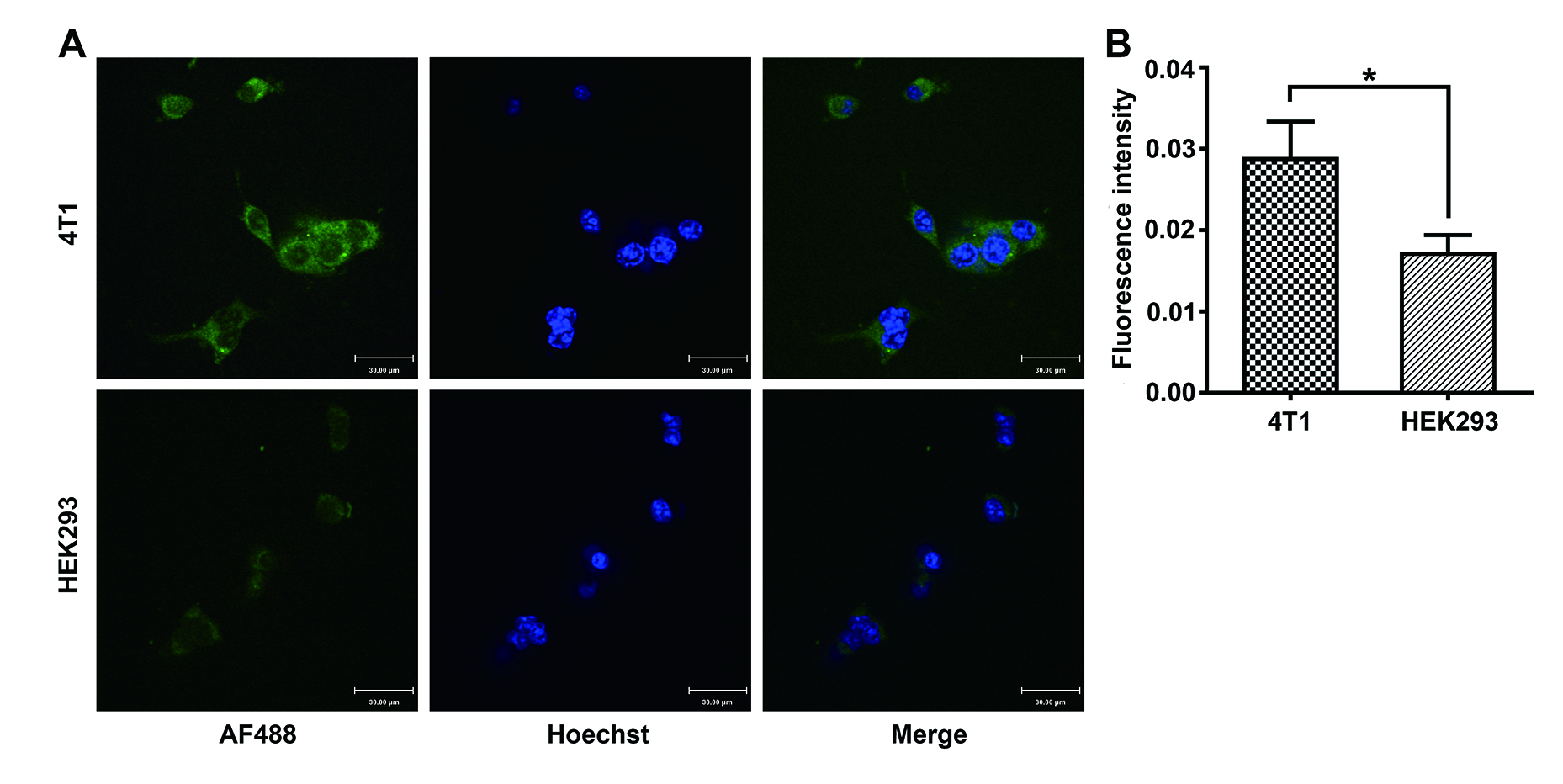

Supplement: Supplementary file 1 — Additional file 1. [file 40644_2021_411_MOESM1_ESM.zip › Supplementary Figure2.tif]

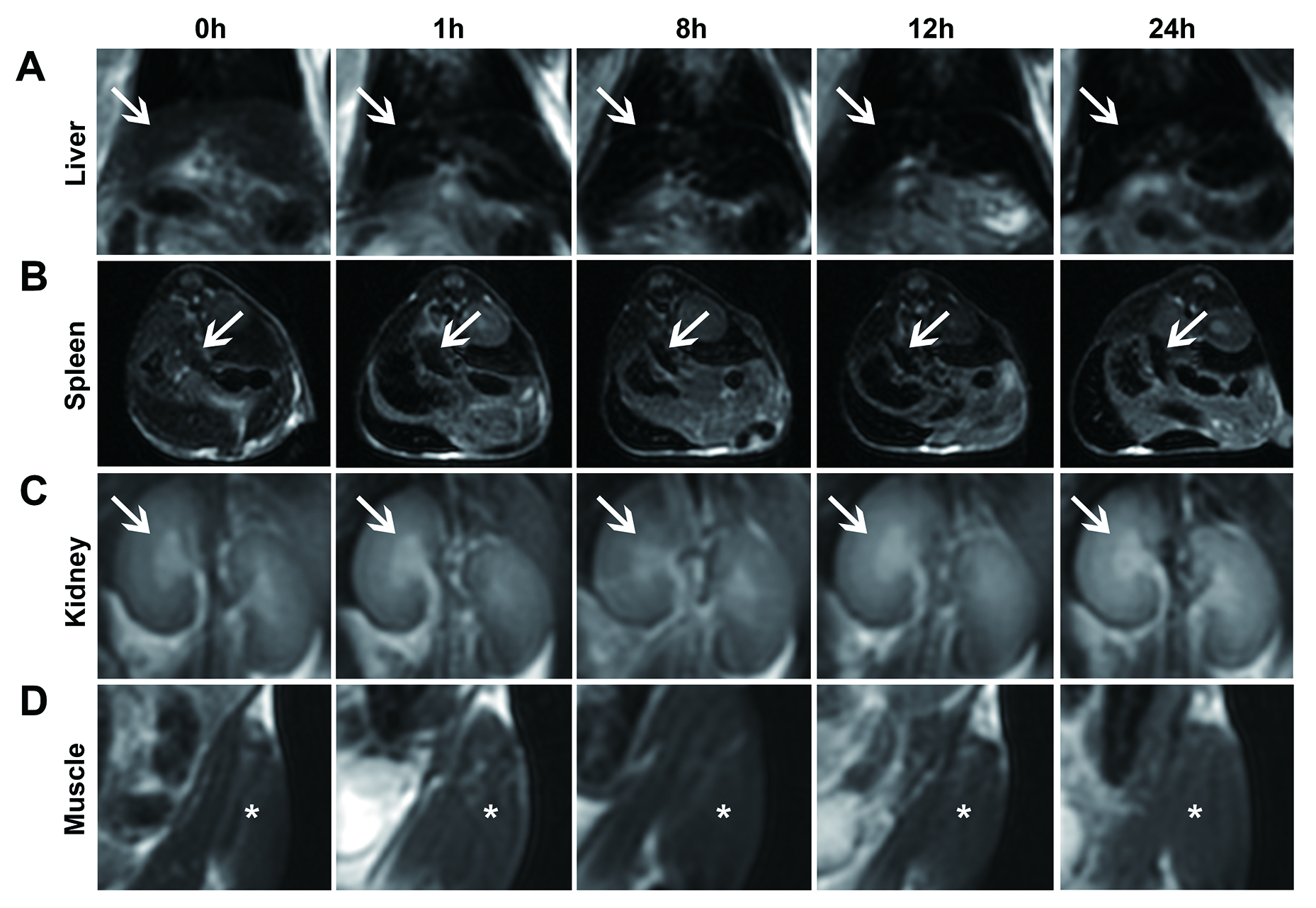

Supplement: Supplementary file 1 — Additional file 1. [file 40644_2021_411_MOESM1_ESM.zip › Supplementary Figure3.tif]

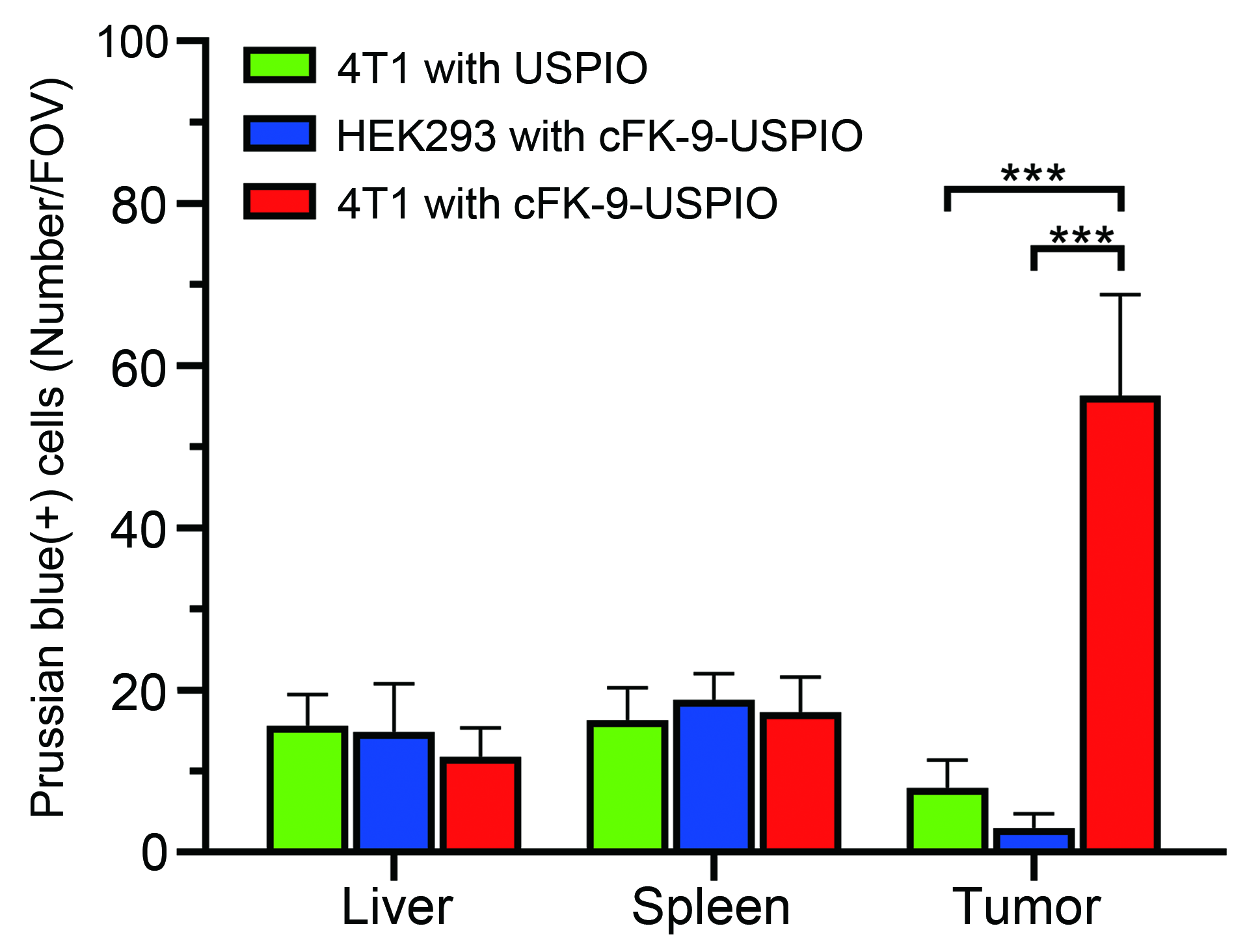

Supplement: Supplementary file 1 — Additional file 1. [file 40644_2021_411_MOESM1_ESM.zip › Supplementary Figure4.tif]

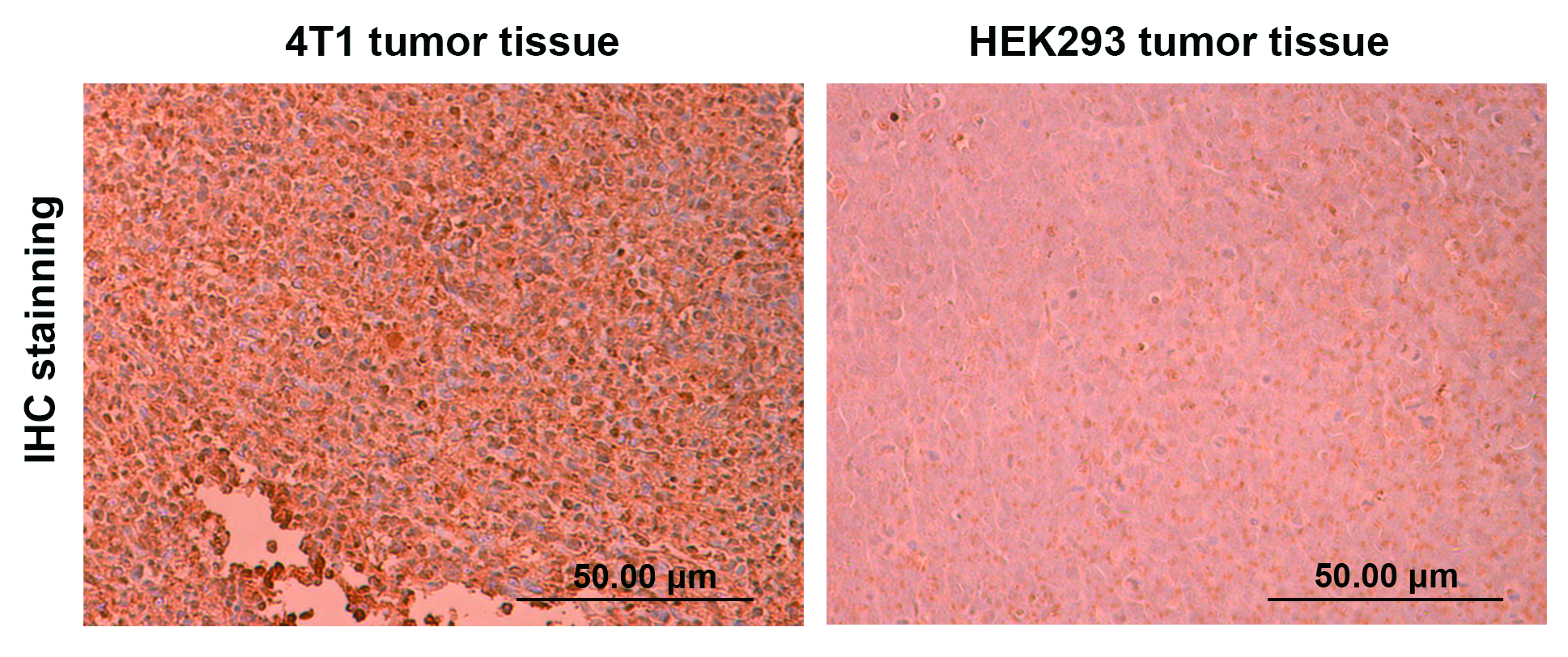

Supplement: Supplementary file 1 — Additional file 1. [file 40644_2021_411_MOESM1_ESM.zip › Supplementary Figure5.tif]
